# The hormetic functions of Wnt pathways in tubular injury

**DOI:** 10.1007/s00424-017-2018-7

**Published:** 2017-07-06

**Authors:** Elisabeth F. Gröne, Giuseppina Federico, Peter J. Nelson, Bernd Arnold, Hermann-Josef Gröne

**Affiliations:** 1 Department of Cellular and Molecular Pathology, German Cancer Research Center (DKFZ), Im Neuenheimer Feld 280, 69120 Heidelberg, Germany; 2 Clinical Biochemistry, Ludwig Maximilian University, Munich, Bavaria Germany

**Keywords:** Wnt pathways, Dkk3, Fibrosis, Atrophy

## Abstract

Chronic tubulointerstitial damage with tubular epithelial atrophy and interstitial fibrosis is the hallmark of chronic kidney disease (CKD) and a predictor for progression of CKD.

Several experiments have now provided evidence that the Wnt signaling pathways are significantly contributing to atrophy and fibrosis; in contrast, it also has been shown that the Wnt system fosters regenerative processes in acute tubular injury.

We now have demonstrated that Dickkopf 3 (DKK3) is an agonist for canonical Wnt signaling in CKD and fosters chronic fibrosing inflammation of the tubulointerstitial compartment. Genetic- and antibody-mediated inhibition of DKK3 leads to a pronounced improvement of tubular differentiation and a reduction in fibrosis.

In addition, the secreted glycoprotein DKK3 can be used as a non-invasive urinary marker for the extent of CKD in man.

## Definition of acute and chronic tubular injury in the study of Wnts

As the effects of Wnt pathways in tubular injury will be discussed, these actions should be put into context of the quality and extent of epithelial injury which may be species-specific and may not necessarily reflect the pathohistology and pathophysiology of acute and chronic renal tubulointerstitial diseases seen in man.

In man, *acute injury of tubular epithelia* can be characterized by morphologic, biochemical, and functional means. In proximal tubules, lesions can vary from slight cytoplasmic vacuolization and a change in brush border height to cell death. Different degrees of loss of cell polarity and differentiation can be associated with emergence of embryonal gene and protein patterns. Exemplarily, vimentin—by convention assumed to be a mesenchymal cytoskeletal protein marker—can be observed temporarily. Once the causative factor ceases to exert its influence on injured epithelia, a regenerative process occurs which may lead to a “restitutio ad integrum”, but can also result in focal chronic atrophic and fibrotic areas of the tubulointerstitium of the cortex and the outer medulla. Cells and matrix surrounding the tubules exert a significant influence on the extent of injury and its recovery. Tubulointerstitial crosstalk is a term to describe the interaction between tubular epithelia and interstitial cells although it obfuscates the lack of knowledge of this process. There can be major differences between rodent models of acute tubular injury and the morphology of acute epithelial lesions seen in human renal biopsies.

In acute renal failure in humans, tubular epithelial injury often occurs without significant inflammatory mononuclear cell infiltrate in the interstitium and without necrosis/ apoptosis of the epithelia. In contrast, in mice, neutrophilic granulocytes populate the peritubular capillaries and the interstitium in acute hypoxic injury.

In order to achieve analogies between mouse and man, animal models may be taken akin to the morphology of acute tubular injury in man.

**Fig. 1:**
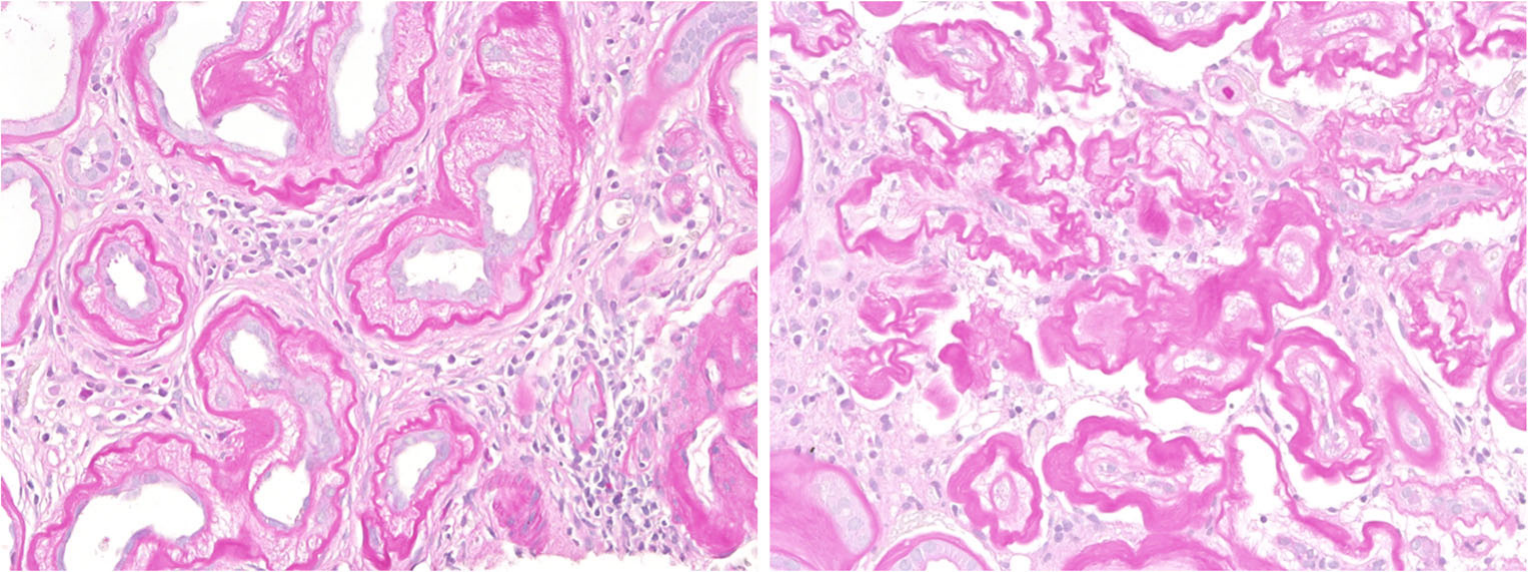
Two significantly different types of interstitial fibrosis and tubular atrophy are shown in human renal biopsies. In the *left* photograph, tubular cells contribute to extracellular matrix increase by a dramatic broadening of the tubular basement membrane. In the *right* photograph, tubular epithelial atresia with skeleta of tubules is surrounded by interstitial matrix produced by interstitial myofibroblasts. Lesions depicted to the *left* of Fig. 1 are not seen in mouse, and lesions as seen to the *right* are seldom observed in mouse (stain: PAS, magnification: 200x)

In *chronic tubular injury*, generally accepted morphologic features have not been defined to which extent an epithelium has to show broadened basement membranes, loss of polarity, and “dedifferentiation” to be irreversibly damaged and termed “atrophic” and “atretic”. The epithelial changes may take place in conjunction with an increase in interstitial matrix or fibrosis. Interstitial fibrosis is thought to be mainly generated by the interstitial myofibroblasts—influenced by fibrogenic cytokines, secreted by tubular epithelia and microvascular endothelia. In diabetic nephropathy though, as well as in other chronic glomerular diseases, degenerating tubules may demonstrate massively broadened basement membranes which can coalesce and contribute significantly to the buildup of interstitial extracellular matrix (Fig. 1). In contrast to the usual association of tubular atrophy and tubulointerstitial fibrosis, chronic tubular injury may develop without a relevant interstitial matrix increase in cases of selectively decreased perfusion, pressure, and glomerular filtration resulting in “inactivity atrophy” of tubules, which often is reversible after restoration of glomerular filtrate. Some animal models may not mirror the slowly progressing tubular degeneration observed in man. As an example of a misguided animal experiment, “damage and interstitial fibrosis” has been studied 5 to 7 days after unilateral ureter obstruction when chronic tubular injury has not yet developed, and interstitial fibrosis—which is not the main characteristic of this model—can hardly be seen.

## Wnt pathways

Wnt is a conjugation of two terms: *W*g, meaning wingless, and I*nt*. Mutation of the wingless gene leads to flies without wings. The Int1 gene enhances breast carcinoma development in mice when the mouse mammary tumor virus (MMTV) integrates into the vicinity of the Int1 gene [2, 3].

There are 19 Wnt ligands (MW≈40kDA) binding either to a heterodimeric complex of lipoprotein receptor related protein 5 or 6 (LRPP 5 or 6) and a member of a seven transmembrane protein Frizzled (Fz)-family with 10 receptor proteins or to receptor tyrosine kinase-like orphan receptor (ROR) or to a receptor-like-tyrosine kinase (Ryk) [5, 7, 12, 26, 32, 38, 39].

The binding of a Wnt to a receptor depends on acylation of Wnt by action of porcupine enzyme and CoA-palmitoleic acid [21, 28, 49]. LRP 5, 6 /Fz can lead to canonical pathway activity with binding of ß-catenin to the transcription factors TCF/ LEF displacing the coinhibitor Groucho [1]. Some target genes are Axin 2, Dickkopf (DKK) 1 and 2, Wnt1-inducible signaling pathway proteins (WISPs), and secreted Frizzled-related proteins (sFRP).

Specific Wnt ligands preferentially lead to activation of the calcium pathway with increased activity of phospholipase C, calcineurin phosphatase, calcium/calmodulin-dependent kinase II (Cam kinase II), protein kinase C, and NFAT transcription factor [2, 3, 15]. In some epithelia, the planar cell polarity (PCP) pathway is activated either by Wnt binding to LRP/Fz or ROR with ensuing activity of Rho and JNK [14].

Multiple factors—some in the plasma membrane, some soluble—can potently activate or inhibit Wnt signaling [12, 22, 49].

In addition to concentration of the ligands/coactivators/coinhibitors, time of activity in a physiologic or pathophysiologic process, duration of activity, and crosstalk with other cytokine triggered pathways are influencing signaling. Also in the kidney, the Wnt pathways are a hormetic system, the effects of which are dramatically different in acute versus chronic tubular epithelial injury models [14, 36, 49]. In order to gain an overview of the effects of Wnt and associated proteins in acute tubular injury versus chronic tubular injury Table 1 lists published data.

**Table 1:** Effects of Wnt pathway-associated proteins in renal tubular injury excluding polycystic kidney disease

Effects of Wnt pathway-associated proteins in renal tubular injury^a^				
Species	Protein	Injury model	Effect	Reference
mouse	Wnt4	IRI	Expressed in proximal tubules, correlated to cyclin a and d1	Terada Y et al. JASN 14:1223, 2003 [40]
mouse	Wnt4	FAT injury	Upregulation	Surendran K et al., Kidney Int 65:2212, 2004 [38]
mouse	secreted Frizzled-related protein 4 (recombinant)	UUO	Inhibition of β-catenin signaling, reduction of myofibroblasts and fibrosis	Surendran K et al., JASN 16:2373, 2005 [37]
mouse	Axin2	IRI 72/120 h	Activation of canonical Wnt (LacZ indicator)	Lin SL et al., PNAS 107:4194, 2010 [18]
mouse	LRP5/LRP6 (LRP5+/−/LRP6+/−)	IRI 72/120 h	Exacerbation of tubular injury in heterozygous deficiency	Lin SL et al., PNAS 107:4194, 2010 [18]
mouse	Wnt2, 2b, 4, 7b, 10a	IRI	Upregulation	Lin SL et al., PNAS 107:4194, 2010 [18]
mouse	β-catenin/CBP (inhibitor ICG-001)	UUO	Reduction of fibrosis	Hao S et al., JASN 22:1642, 2011 [9]
mouse	expression of Klotho (plasmid injection)	UUO (7 days) adriamycin nephropathy (3 weeks)	Reduction of interstitial fibrosis, tubular dilation and fibrosis inhibition by inhibition of β-catenin	Satoh M et al., Am. J. Physiol 303F1641, 2012 [33]
mouse	β-catenin−/−	IRI (1 day) FAT injury (2 days)	Exacerbation of tubular injury in heterozygous deficiency	Zhou D et al., Kidney Int 82:537, 2012 [47]
mouse	Dapper 3 KO (negative regulator of Wnt)	UUO	Increase in renal fibrosis	Xue H et al., JBC 288:15,006–15,014, 2013 [43]
mouse	Wnt (loss of Klotho)	UUO	Increase of fibrosis by Wnt signaling	Zhou L et al., JASN 24:771, 2013 [48]
mouse	secreted Frizzled-related protein 1 KO	UUO	Increase in interstitial fibrosis markers and epithelial dedifferentiation by non-canonical Wnt signaling	Matsuyama M et al., JBC 289:31,526–31,533, 2014 [23]
mouse	Wnt/β-catenin indicator mice	Acute IRI (30–75–60 min) glycerol-induced acute kidney injury (3 days, 6 days)	Tubular cell regeneration restricted to epithelia with Wnt signaling	Rinkevich Y et al., Cell Rep 4:1270–1283, 2014 [31]
rat	Agonist of canonical Wnt/β-catenin 2-amino-4 [3, 4-(methylendioxy) benzylamino] 6- (3-methoxyphenyl) pyrimidine	Bilateral IRI (60 min-24 h)	Decrease of serum creatinine and tubular injury	Kuncewitch M et al., Shock 43:268–275, 2015 [16]
mouse	Wnt/β-catenin	Glycerol-induced kidney injury (3 days)	β-catenin/TCF reporter activity in CD24 (progenitor marker) positive injured tubular epithelia	Zhang Z et al., Stem Cells Int. ID391043, 2015 [44]
mouse	Porcupine- (Wnt-acyl transferase) Blocker Wnt-C59	UUO	Blockade of all Wnts, of renal fibrosis, of inflammatory cytokines	Madan B et al., Kidney Int 89:1062, 2016 [21]
mouse	Wnt4	Bilateral IRI (20 min–24 h)	Increase of Wnt4 correlating with tubular injury	Zhao SL Sci Rep 4:32,610, 2016 [45]
rat/mouse	PEDF (pigment epithelium-derived factor)	UUO (5 days)	Increase in interstitial inflammation and rise in interstitial fibrosis	He X et al., Kidney Int 91:642–657, 2017 [11]
mouse	PRR (pro-renin receptor)	IRI adriamycin angiotensin II infusion	High expression of downstream targets such as fibronectin, plasminogen activator inhibitor 1, and α–smooth muscle actin. Worsening of kidney dysfunction, and worsened renal inflammation and fibrotic lesions	Li Z et al., JASN Mar 7, 2017 [17]
mouse	WIs (Wntless, synonym for Evi cargo receptor for Wnts) tubule specific KO	UUO	Reduction of renal fibrosis, myofibroblasts activity	Zohu D et al., JASN Mar 23, 2017 [46]

*IRI* ischemia reperfusion injury, *FAT injury* folic acid tubular injury, *UUO* unilateral ureteral obstruction

^**a**^Excluding polycystic kidney disease

It is apparent that Wnt-associated proteins exert relevant effects, perhaps due to their actions independent of a single Wnt in contrast to the selective action of a specific Wnt.

## Dickkopf 3 (DKK3)

We have focused on the DKK family which can inhibit or activate Wnt pathways, dependent on the microenvironment [8, 22, 27, 35, 42]. One protein out of this family of Wnt-associated proteins, namely DKK3, synthesized by stressed tubular epithelia, has recently been shown by us to significantly modulate chronic tubulointerstitial injury and be of use as a non-invasive urinary marker for chronic tubulointerstitial lesions [6].

The DKK3 family consists of five proteins: DKK1, 2, 3, 4, and a DKK-like protein 1 (DKKL1, also known by the synonym Soggy) which is constitutively expressed in immune-privileged organs in adult mammals, such as the testis, eye, and brain, but not in the adult kidney [6, 27]. DKK 1–4 share two conserved cysteine-rich domains (Cys). Cys1 is an N-terminal domain which is unique for DKKs, not found in other vertebrate proteins. The two Cys are separated by a linker region of variable length which is similar in DKK1, 2, 4, but small in DKK3 with only 13 amino acids.

Studies on DKK1 have demonstrated that DKK1 binds LRPs and inhibits Wnt signaling. DKK2 may have agonistic as well as antagonistic effects on Wnt pathways. Published effects of DKKs on the kidney are summarized in Table 2.

**Table 2:** Effect of DKK-proteins in acute and chronic renal tubular injury

Effect of DKK-proteins in acute and chronic renal tubular injury				
Species	Protein	Injury model	Effect	Reference
mouse	DKK1 recombinant	UUO 7–14 days	Reduction of cMyc, twist, collagen, fibronectin, fibrosis	He W et al., JASN 20:765–766, 2009 [10]
mouse	Recombinant C2 cysteine-rich domain of DKK2 infused	Bilateral IRI (infusion starts at day 0 or 1)	Tubular regeneration and faster fall of serum creatinine	Lin SL, PNAS 107:4194–4199, 2010 [18]
human	SNPs of DKK3 and mRNA DKK3	Adult polycystic kidney disease (ADPKD)	Association with large cysts	Liu M et al., JASN 21:1510–1520, 2010 [19]
mouse	DKK1 AAV-increased circulating DKK1	UUO 4 days/unilateral IRI	DKK1 binding to LRP6 blocking PDGFBB pathway inhibiting inflammation, fibrosis	Ren S et al., PNAS 110:1440–1445, 2013 [30]
mouse/human	DKK3 deficiency	UUO 21 days/adenine nephropathy28 days/human urine	General and tubular specific DKK3 deficiency with reduction of tubular epithelial dedifferentiation and decrease of fibrosis. In man, DKK3 in urine: marker of extent of tubular atrophy and fibrosis	Federico G et al., JCI Insight 1:e84916, 2016 [6]
mouse	DKK1 recombinant	UUO	Inflammation, fibrosis downregulated	Johnson BG et al., JASN 6080826, 2017 [13]

*IRI* ischemia reperfusion injury, *UUO* unilateral ureteral obstruction

DKK3 has different effects as compared to the DKK1, 2, and 4. DKK3 is strongly expressed in mesenchymal progenitor and mesenchymal cells in vitro; it also exerts potent immunosuppressive effects [20, 24, 29]. We have shown that a deficiency of DKK3 led to an exacerbation and significant prolongation of experimental autoimmune encephalitis [29].

Based on our immunologic studies with DKK3 in the skin, brain, and the lymphopoietic system, we have postulated that DKK3 might demonstrate immunosuppressive and fibrogenic actions in chronic renal disease.

For the studies on the effects of DKK3 in CKD, two models of chronic tubular and interstitial damage were chosen to demonstrate the chronic renal effects of DKK3:

1. The unilateral ureter obstruction (UUO) model with a primary biophysical or mechanical impact for a maximum of 21 days and
2. The tubular toxicity driven model of chronic oral administration of adenine for a maximum of 28 days

**Fig. 2:**
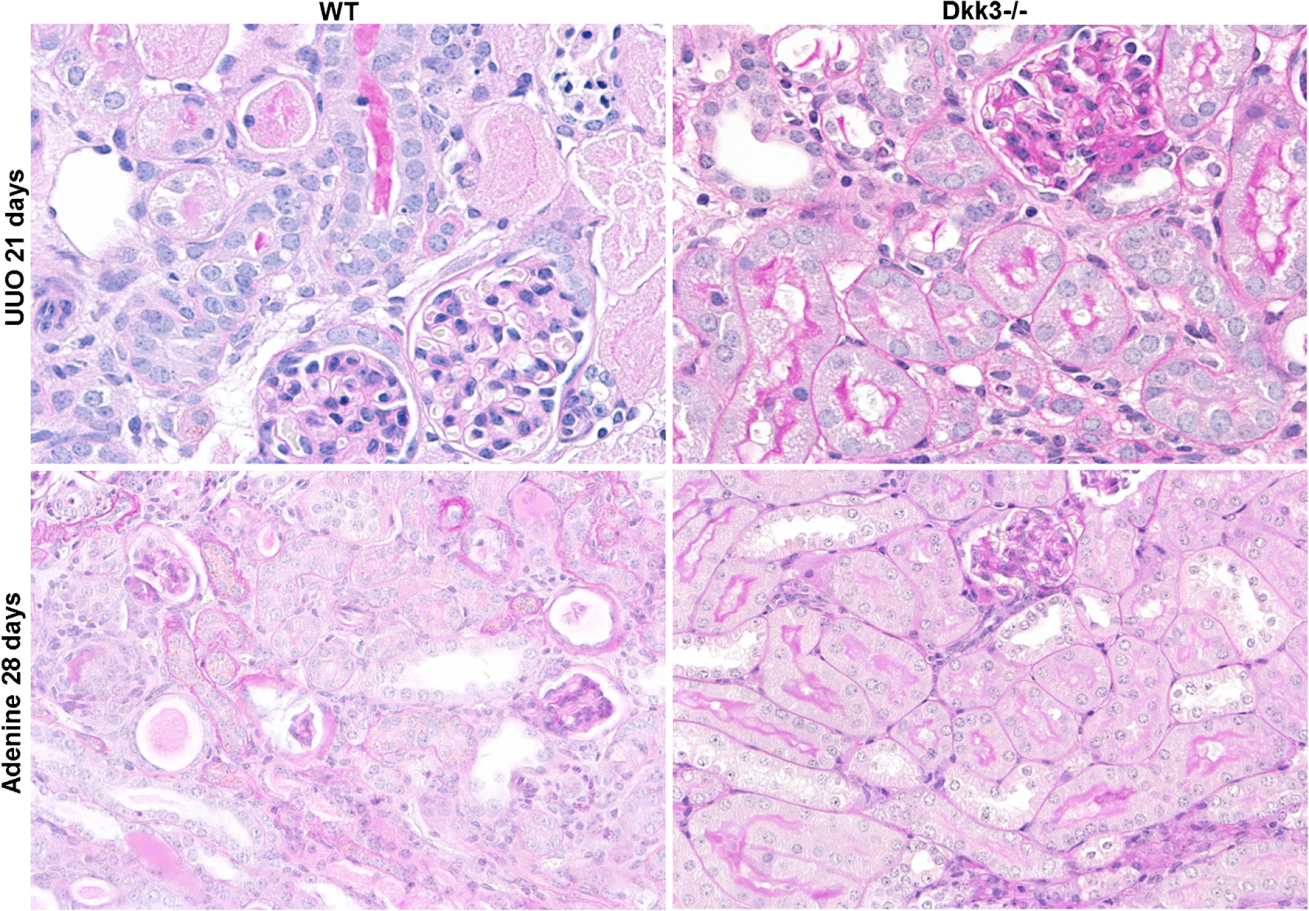
DKK3-deficiency (graphs to the *right*) preserves tubular epithelial differentiation and reduces interstitial fibrosis in two different chronic models of tubulointerstitial fibrosis: UUO (unilateral ureter obstruction) and adenine nephropathy (stain: PAS, magnification: 200x; male C57Bl/6 mice 8 to 12 weeksold were used; adenine was administered in food at a concentration of 0.25%)

In both models, a systemic knockout of DKK3 lessened tubular dedifferentiation and interstitial matrix increase (Fig. 2). This may seem surprising as in adult mice DKK3 has not been detected in the kidneys as documented by the DKK3-promoter-driven enhanced green fluorescence protein (eGFP) transgene mouse. In injured tubular epithelia though, DKK3 was demonstrated. Our starting hypothesis had been that as DKK3 is expressed during development it shall be re-expressed in pathophysiologic processes due to a hitherto unexplained general mechanism by which an embryonal pattern can be re-expressed at least partially in pathologic processes.

In order to provide evidence for the major DKK3 synthesizing cell, we have taken different approaches. A tubular and collecting duct epithelia-specific DKK3-deficiency—achieved by crossbreeding Pax8-Cre mice to a mouse line with a floxed DKK3 gene—also led to a pronounced preservation of renal parenchyma in both, the mechanical as well as the toxic model. This approach has provided evidence that DKK3-deficiency in tubular epithelia impeded tubular atrophy. This result has not been expected as regenerating cells have been associated with an active Wnt pathway, perhaps even taking place in differentiated cells [31, 41]. Injection of pure T cells from wild-type mice and DKK3-systemically deficient mice into T cell-deficient Rag2 mice caused an increase in chronic tubulointerstitial injury in UUO to a similar degree as observed in wild-type mice, excluding T cells as the DKK3 synthesizing cell population for the prevention of chronic tubulointerstitial injury in DKK3 deficiency. Genetic approaches may have the drawback of induction of compensatory genetic mechanisms. Therefore, we have applied a long lasting blockade of DKK3 by repetitive injection of a functionally antagonistic DKK3 antibody. A significant reduction of tubular lesions and interstitial fibrosis could be observed by DKK3-antibody therapy [6].

**Fig. 3:**
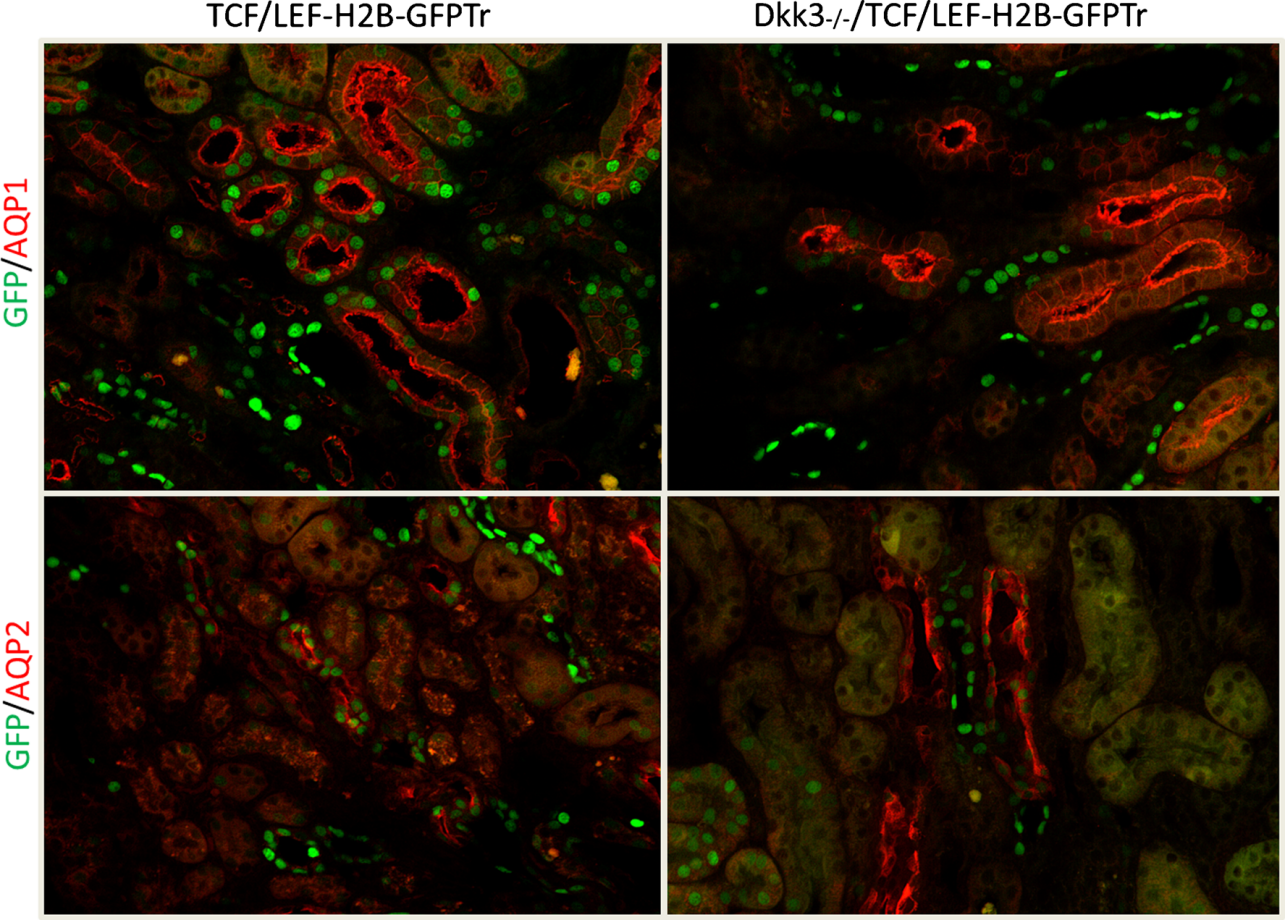
“Canonical Wnt signaling” indicator mice demonstrate evident nuclear fluorescence in wild-type mice with UUO after 7 days; in contrast, DKK3 deficiency mice have a significant reduction in renal tubular epithelial fluorescence (AQP1: aquaporin 1 for proximal tubules, AQP2: aquaporin 2 for collecting ducts, TCF/LEF-H2B-GFPTr: reporter mouse with expression of green fluorescent protein when promoter regions of the Wnt dependent transcription factors TCF/LEF are activated by the canonical Wnt pathway. As nuclear fluorescence is evaluated unspecific background stain does not interfere)

The cell-molecular sequence interrupted or at least partially impeded by DKK3 inhibition has been analyzed by RNA-next generation sequencing, T cell transcription factors (GATA3 and Tbet), and cytokine measurements. The Th2 response in wild-type mice with UUO switched to a Th1 T cell activity with an increase in IL1ß, IFNγ, and TNFα. Specifically elevated INFγ concentrations have been associated with a suppression of increased matrix production; one potential mechanism is the repressive action of INFγ on transcription of collagen genes. A regulatory component to this increased Th1 T cells was an increase in Foxp3 regulatory T cells infiltrating DKK3-deficient kidneys in higher quantities than in wild-type kidneys. These experiments also have established that a controlled “acute” inflammation might prevent chronic fibrosing lesions. In addition, experiments with indicator mice for the canonical Wnt pathway have suggested that DKK3 activates this Wnt signaling mode (Fig. 3). We have not been able to depict whether DKK3—a secreted glycoprotein—induces tubular epithelia to secrete fibrogenic cytokines in an autocrine manner or whether DKK3 acts directly on T cells and/or interstitial fibroblasts to induce interstitial matrix deposition [4, 14, 25, 34].

The secretion of DKK3 has been used to evaluate chronic tubulointerstitial changes in mouse and man by measuring concentration and quantity of DKK3 in urine. We have determined urinary DKK3 concentrations in two different age groups: children/adolescents and adults, both with mixed etiologies of their chronic renal diseases. The urine DKK3 ELISA-measurements have been correlated to morphometrically determined tubular atrophy and interstitial fibrosis in renal biopsies in the adult population and to proteinuria, plasma creatinine, and estimated GFR (e-GFR) in the pediatric and adult cohorts ranging in number from 32 (adult) to 72 (pediatric). There was no correlation to proteinuria. ROC (Receiver-Operating-Characteristic) curve established a significantly higher correlation of the DKK3 values to tubular atrophy and to interstitial fibrosis—regarded as two separate entities—than of the serum creatinine concentration and e-GFR [6].

Although the clinical value of DKK3 urinary measurements for the determination of tubular atrophy and interstitial fibrosis has to be corroborated in larger and ethnically divergent patient cohorts, we are convinced that a non-invasive urinary biomarker for chronic tubulointerstitial damage, relating to prognosis and course of the chronic renal disease, has been detected.

In summary, there is ample evidence that the Wnt pathways play a decisive role in the regenerative or reparative processes after acute injury and in the progression of chronic tubular injury. Several issues remain unresolved:

1. Which factor (s) determine that Wnts act in a regenerative manner in acute tubular injury and in contrast act in a perpetuating and exacerbating manner in chronic tubular injury?
2. Which Wnts and associated proteins and which Wnt pathways are dominant in acute and in chronic injury and may this depend on the kind of injury?
3. With which other pathways do Wnts interact or even exert their major action?
4. Do Wnt-associated proteins solely act by Wnt or do they exhibit multivalency? LRPs can interact with other pathways [14, 32].
5. Which is (are) the interacting protein (s), lipid (s), or receptor(s) for some of the Wnt-associated proteins? (e.g., for DKK3 neither a receptor nor a direct interacting protein has been unambiguously detected).
